# Simple technique of subxiphoid hernia correction carries a low rate of early recurrence: A retrospective study

**DOI:** 10.1186/s12893-017-0249-3

**Published:** 2017-05-05

**Authors:** Gustavo Heluani Antunes de Mesquita, Leandro Ryuchi Iuamoto, Fabio Yuji Suguita, Felipe Futema Essu, Lucas Torres Oliveira, Matheus Belloni Torsani, Alberto Meyer, Wellington Andraus

**Affiliations:** 1 Department of Gastroenterology, University Of São Paulo Medical School, Av. Dr. Arnaldo, 455 - Cerqueira César, São Paulo, SP 01246-903 Brazil; 2 Abdominal Wall Repair Center, Samaritano Hospital, São Paulo, Brazil; 3 Division Chief, General and Gastrointestinal Surgery, Dante Pazzanese Institute of Cardiology, São Paulo, Brazil

## Abstract

**Background:**

Subxiphoid incisional hernia occurs as a complication following median sternotomy and are difficult to repair. We present recent data of a standardized technique for correction of subxiphoid incisional hernias, and discuss possible anatomical and surgical factors related to recurrence of the hernia.

**Methods:**

A retrospective study with medical records analysis of patients submitted to surgical correction of subxiphoid incisional hernias through standardized treatment between July 2014 and September 2016. All procedures were carried out using the same standardized technique, surgical materials (threads and meshes) and pre- and post-operative care.

**Results:**

All of the surgical procedures carried out were elective. The hernia defect varied between 5 cm and 16 cm (mean of 7.4 cm); the procedure lasted between 32 and 75 min; the mean time of hospital stay was 2.2 days (range from 1 to 5 days). In five patients the correction of subxiphoid incisional hernia was carried out concurrently with another procedure. No death occurred as a result of the operations. Five patients had minor postoperative complications. Follow up time was between 7 and 33 months, with a recurrence rate of 0% at the time of writing.

**Conclusions:**

Despite the limitations of a short follow up period, the surgical technique described presented low rates of early recurrence by closing the hernia defect, using relaxing incisions in the musculature and aponeurosis and surgical mesh.

## Background

Cardiovascular surgeries are extensive procedures which patients are submitted to several types of morbidity during surgery and on post-operative period [1]. Sternotomy’s problems depend on surgery techniques employed as well as patients overall health condition.

Subxiphoid incisional hernia is a complication following median sternotomy, such as myocardial revascularization, cardiac valvuloplasty and cardiac transplantation. The incidence rate of subxiphoid incisional hernia is between 1% and 4.2% [2, 3], although the true incidence rate is unknown because this hernia is generally asymptomatic. Therefore, patients do not seek medical assistance and the hernia is underreported.

The development of subxiphoid incisional hernias following sternotomy is related to several factors, such as obesity, infection of the operative wound, patients under immunosuppression therapy, patients receiving transfusions during surgery [4].

The definitive treatment of subxiphoid incisional hernia is the surgical closing of the abdominal fascia [5, 6]. For this, different methods may be used, such as the sole use of suturing, the use of surgical mesh in open surgery or laparoscopy. The positioning of the mesh may be carried out through a variety of methods such as onlay, sublay, sublay with another mesh adjusted to the hernia defect, or even intraperitoneal. [3–7]. The techniques of closing the abdominal fascia, the type of suture utilized, the spacing between the edge of the hernia and the edge of the surgical mesh vary greatly in the published literature, and even within the proceedings of the same study, in the absence of standardized treatment with proven efficiency.

The recurrence rate and complexity of the hernia repair are high, due to the anatomy of the affected area and lateral traction generated by respiration and coughing [3]. As well as the wide variation in recurrence rates, the follow up time of patients is insufficient. When Mackey et al. [8] followed up patients for 27 to 69 months, the recurrence rate was 43% from exclusive use of sutures, 33% from use of surgical mesh in open surgery, and 30% from the placing of mesh using surgical laparoscopy, and in 75% of patients with an infection of the sternal wound.

Losanoff et al. [3] also found in a review that the recurrence rate from 4 to 69 months was between 43% and 80% for the exclusive use of sutures, and between 0% and 33% for use of mesh in open surgery. The use of laparoscopic surgery to place surgical mesh [9], on the other hand, had a recurrence rate of 10% over a follow up period of 20 to 42 months.

The majority of studies are very outdated, especially those with a higher number of patients included. There is no standardized treatment for subxiphoid incisional hernias, technical details of the surgery or follow up periods. This means that reported rates of recurrence are highly variable.

Therefore, our objective is to present recent data of a standardized technique for correction of subxiphoid incisional hernias, and highlight possible anatomic and surgical factors related to recurrence of the hernia.

## Methods

A retrospective study with analysis of the medical records of all patients submitted to surgical correction of subxiphoid incisional hernias through standardized treatment between July 2014 and September 2016, in Dante Pazzanese Institute of Cardiology, Sao Paulo, Brazil. The hernias were diagnosed through history and detailed physical examination in walk-in centers. The follow-up was made with medical appointments after 1 week, 1 month, 3 months, 6 months and annually after surgery.

A descriptive analysis was used to compare parameters such as age, sex, comorbidities, body mass index (BMI), smoking status, the cause of sternotomy, history of surgical procedures and associated surgical procedures. The circumstances of admission for the subxiphoid hernia correction surgery (elective or emergency) were also compared, as were the medications administered.

The analysis also compared surgical parameters such as the size of the hernia, the time in surgery, the time of postoperative hospital stay, complications, follow up time and rate of recurrence.

The study was approved on Dante Pazzanese’s ethical committee and informed consent was obtained from all individual participants included in the study.

### Surgical technique

All procedures were carried out using the same standardized technique, surgical materials (threads and meshes) and pre- and post-operative care.

**Fig. 1:**
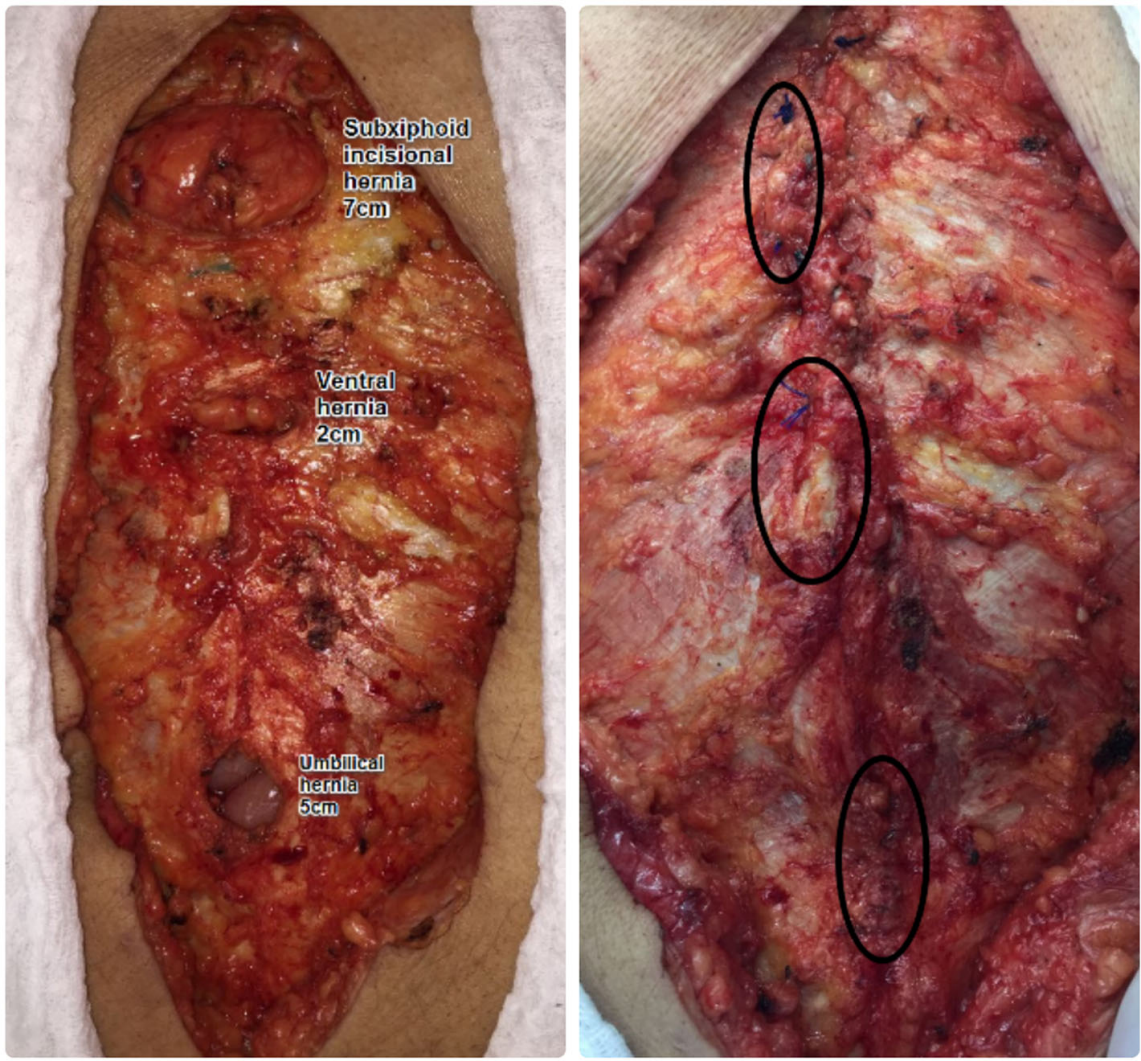
On the left picture, resection of the hernia sac and reduction of the content to the abdominal cavity. On the right, continue sutures closing the abdominal fascia in two planes, using non absorbable thread

**Fig. 2:**
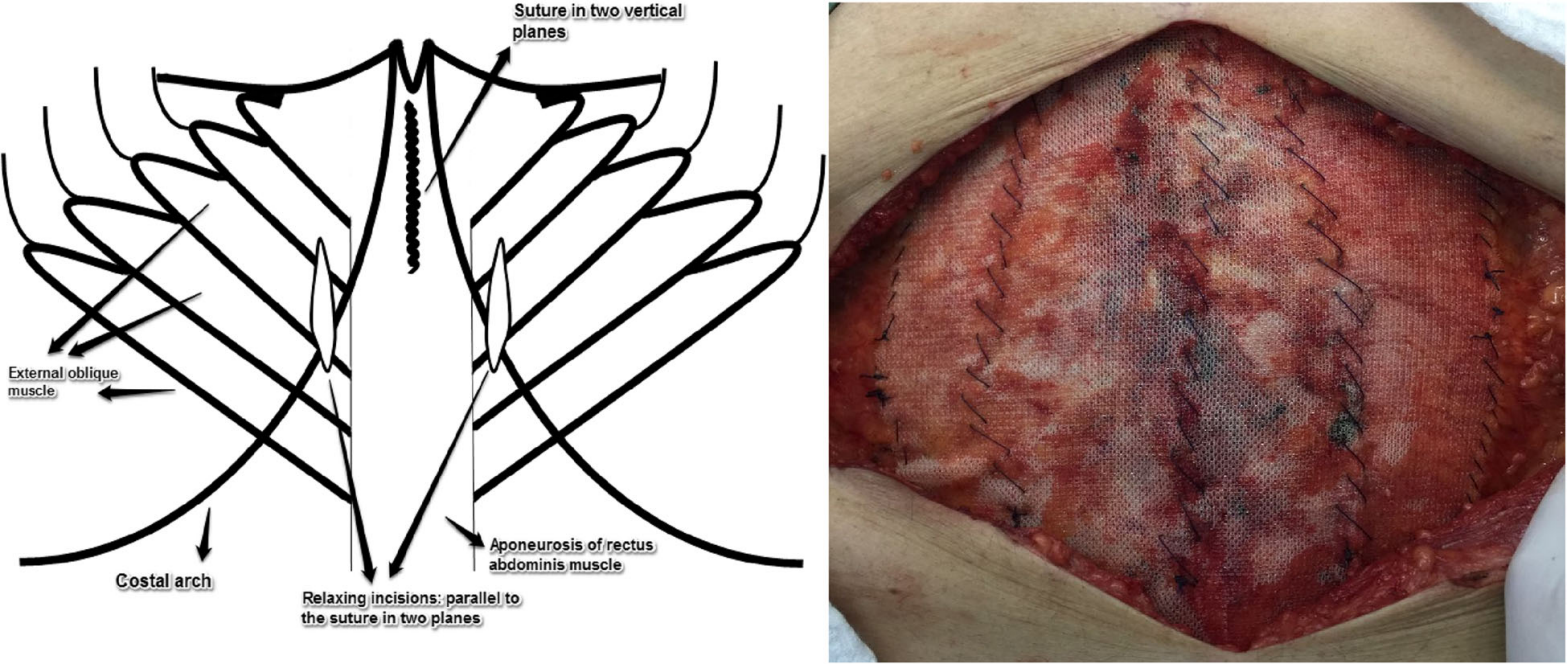
On the right, illustration showing the closure in two vertical planes and the relaxing incisions. Detail on mesh fixation: continuous suture was applied around the edge of the mesh using absorbable thread. Complementary sutures were also applied

1. Antibiotic prophylaxis was administered at anaesthetic induction (cephalosporin 1 g).
2. Incision and resection of the previous scarring.
3. Debridement of the hernia and resection of the hernia sac.
4. Reduction of the hernia contents for the abdominal cavity. (Fig. 1)
5. Closing in two vertical planes of the hernia defect, one above the other, through all the abdominal wall layers in each of them, with a continuous suture using nonabsorbable thread (Prolene® 1.0, Johnson & Johnson). (Fig. 1 and Fig. 2)
6. Vertical relaxing incision, parallel to the hernia defect closure, in the musculature and aponeurosis for approximation of the hernia edges when necessary. (Fig. 2)
7. Placement of a Marlex onlay mesh (weight 120 +/− 10 g/m^2^, pores 0.9 +/− 0.1 mm) (Abdotex®, Barone, Campinas-SP, Brazil) with at least 5 cm of longitudinal extension and 10 cm of transversal extension in relation to previous defects.
8. Fixing of the mesh with a continuous suture using absorbable thread (Vicryl® 2.0, Johnson & Johnson) around the edge of the mesh. Other continuous sutures for adequate fixing and mesh incorporation are also applied. (Fig. 2)
9. A vacuum closed drain is applied for three days, or until drainage is less than 100 ml/day.

## Results

The study included 15 patients. Nine (9) were male and six (6) female, with a mean age of 62.65 years, and a mean BMI of 29.6 (range of 20.7 and 43.11). Among the patients, 73,3% presented more than one comorbidity and 40% had a history of smoking. All comorbidities found are shown in Table 1, as well as a detailed profile of each patient. All patients were under full anticoagulation with low molecular weight heparin, which was not suspended for surgery.

**Table 1:** Shows the detailed comorbidity profile of each patient, and the incidence of each comorbidity in the total of patients

Patient number	Obesity	Smoking history	COPD	Type 2 diabetes mellitus	Imunossupression	Rheumatism	Congestive cardiac failure	Dyslipidemia	Atrial fibrillation	Hypothyroidism	Chronic renal insufficien-cy	Thrombo-cytopenia	Single kidney	Systemic arterial hypertension	Coronary artery disease
1	x					x									
2	x	x		x										x	
3	x			x				x						x	x
4		x										x		x	
5		x	x					x						x	
6															
7	x	x						x		x				x	x
8						x									
9							x	x							x
10	x							x						x	x
11															
12	x	x		x				x					x	x	
13						x									
14	x	x	x											x	x
15					x		x		x		x			x	
Percentage	46,60%	40%	13,33%	20%	6,67%	20%	13,33%	40%	6,67%	6,67%	6,67%	6,67%	6,67%	60%	33,30%

The cause of previous sternotomy was cardiac valve surgery in 46.6% (7) of patients. Of these, six patients had a history of more than one cardiac valve surgery. Six patients (40%) of patients had a history of myocardial revascularization; one patient underwent a change of valve in conjunction with vascularization and one underwent a cardiac transplant.

All subxiphoid hernia corrections were elective, but two were carried out together with incarcerated hernias correction, one inguinal and the other umbilical. In five patients the correction of subxiphoid incisional hernia was carried out concurrently with another procedure; correction of inguinal hernia (1), correction of umbilical hernia (1), cholecystectomy (1) and removal of metallic thread (2).

As a result of the surgical parameters, the hernia defect (longitudinal length) varied between 5 cm and 16 cm, with a mean of 7.4 cm; the procedure lasted between 32 and 75 min; the mean time of hospital stay was 2.2 days (varying between 1 and 5 days). Follow up time was between 7 and 33 months, with a recurrence rate of 0% at the time of writing. No drop out was registered. With regards to complications, there were two hematomas of the operating wound and three operating wounds with partial dehiscence, closed at a second attempt. All complications were resolved conservatively.

## Discussion

Subxiphoid incisional hernia is a complication following the procedure of sternotomy that can carry high rates of recurrence (up to 80%) if not appropriately corrected [3]. In our literature review and surgical practice, it is clear that the use of an appropriate technique is essential to reduce rates of recurrence, which in our study was 0% over a follow up period of 7 to 33 months.

The patients included in this study presented various cardiac diseases, such as cardiac valve failure, congestive cardiac failure and coronary artery disease. These were for the most part older patients (mean age of 62.65 years), and with an elevated BMI, many either overweight or obese (13 patients).

Other studies [8–10] presented a similar profile of patients, with patients with cardiac diseases, the elderly and those with elevated BMI predominating. One Korean study [10] presented a mean BMI score lower this study and others. Correlation was also found with patient comorbidities, with a high prevalence of systemic arterial hypertension, dyslipidemia and diabetes. This study, however, also found a high incidence of rheumatism (20%), congestive cardiac failure (20%), chronic obstructive pulmonary disease (13.3%), atrial fibrillation (13.3%), which was not found, or found in much lower rates in other studies analyzed [8–10]. None of these factors, together or in isolation, influenced variations in technique, rates of recurrence, operating time or complications.

Techniques for incisional hernia correction without closing the fascia have already been described in the literature [4–7], with only the placement of the surgical mesh to cover the opening in the abdominal fascia [11]. The separation of the edges of the aponeurosis in the early postoperative phase was associated with the development of incisional hernias [12]; all cases that presented a distance of greater than 12 mm between the edges of the aponeurosis developed hernias [13]. Therefore this technique is questionable as a method of incisional hernia correction, because it does not close the hernia defect, it only blocks it partially and temporarily. This results in the herniation of the abdominal contents.

With non-closure of the aponeurosis [5], the criteria for defining hernia recurrence becomes debatable, as the hernia defect remains open. Landau et al. [9], for example, had recurrence rates (10%) lower than the majority of studies, however in the surgical technique, closure of the abdominal fascia was not carried out, and therefore the criteria to assess whether or not the hernia was recurrent was unclear. To avoid the occurrence or recurrence of incisional hernias, the line of the suture should be able to hold the edges of the aponeurosis together in position for the duration of the postoperative period [14]. We decided to close the abdominal fascia with a continuous suture and nonabsorbable thread to guarantee greater effectiveness in the closure and reduce the risk of recurrence.

In the closure of hernia defects with sutures [6], the proximity of the edges of the defect can generate excessive pressure, causing dehiscence. This explains the high rate of recurrence when only sutures are used in repair of subxiphoid incisional hernia repair [3, 8]. To avoid dehiscence the use of surgical mesh is advised, which helps scarring to promote a local inflammatory process which induces the production of collagen [15]. However, the mesh alone does not promote the closing of the abdominal fascia; it is not an effective strategy to correct hernias in itself. Its role should be in conjunction with suturing of the hernia defect, as it was with the patients in our study.

Strategies like carrying out relaxing incisions in the musculature of the aponeurosis are essential for the correct closure of the abdominal fascia, because they reduce lateral traction in the epigastric region and allow better approximation of the edges of the hernia defect. With the approximation with the hernia edges, the suturing of the abdominal fascia becomes more effective, and the risk of dehiscence is reduced. Lastly, the use of surgical mesh guarantees the stability of the closing and reduces the risk of dehiscence.

The combination of the closure of the hernia defect, the use of relaxing incisions in the musculature and aponeurosis and the use of surgical mesh could be identified as the factors that led our study to a lower rate of recurrence than the others. In the literature [16] an estimated 35.19% of recurrence in incisional hernias occurs in the first year, 55.72% in the second year. In our study, all patients were followed for at least six months, and 60% of patients were followed up for over a year, with a recurrence rate at the time of writing of 0%. Despite the limitations of a short follow up period, these data are a good predictor of long term lower recurrence rates using the described technique because a large proportion of recurrence occurs up to a year after surgery [16].

As well as the results referring to recurrence, our study also showed a shorter mean hospital stay time than the majority of other studies [6, 7, 15], varying between 1 and 5 days, with a mean of 2.2 days. The shorter mean postoperative hospital stay in our study could be explained by the lower complication rates, quicker recuperation, and a higher success rate in surgery.

The recurrence rate in our study will be analyzed over longer follow up periods to demonstrate that this technique continues to display these lower rates of recurrence. With longer follow up it will be possible to demonstrate that the technique is an acceptable standard for surgical correction of subxiphoid incisional hernia.

## Conclusion

The surgical technique described presented low rates of early recurrence by closing the hernia defect, using relaxing incisions in the musculature and aponeurosis and surgical mesh. Further studies with longer follow-up time should confirm these findings.
